# Comprehensive Analysis of Genetic and Morphological Diversity in *Echinochloa* spp. Populations Infesting Paddy Fields in Ningxia, China

**DOI:** 10.3390/ijms26125623

**Published:** 2025-06-12

**Authors:** Jinhui Li, Yi Zhang, Yan Liu, Shouhui Wei, Zhaofeng Huang, Lu Chen, Hongjuan Huang

**Affiliations:** 1 State Key Laboratory for Biology of Plant Diseases and Insect Pests, Institute of Plant Protection, Chinese Academy of Agricultural Sciences, Beijing 100193, China; ljh981126@163.com (J.L.); shwei@ippcaas.cn (S.W.); huangzhaofeng@caas.cn (Z.H.); 18247464761@163.com (L.C.); 2 Institute of Plant Protection, Ningxia Academy of Agriculture and Forestry Sciences, Yinchuan 750002, China; nxzhy0951@163.com; 3 Institute of Environment and Plant Protection, Chinese Academy of Tropical Agricultural Sciences, Haikou 570100, China; liuyanzb1226@163.com

**Keywords:** barnyard grass, genetic diversity, morphological traits, SCoT marker, DNA barcode, cluster analysis

## Abstract

Barnyard grass is the most problematic weed in paddy fields in Ningxia. Its substantial morphological variation complicates both identification and control, yet the genetic diversity of barnyard grass infesting paddy fields in Ningxia has not been thoroughly studied. In this research, we analyzed the genetic diversity of 46 barnyard grass populations from Ningxia’s paddy fields based on the assessment of morphological traits, DNA barcoding, and SCoT-targeted gene markers. Nine morphological traits were quantitatively analyzed, among which three phenological traits, i.e., leaf length, stem diameter, and plant height, exhibited notable variations. Correlational analysis revealed a positive relationship between morphological traits and multi-herbicide resistance profiles. To assess genetic diversity, four DNA barcodes (*ITS*, *psbA*, *matK*, and *trnL-F*) were used, among which *ITS* demonstrated the strongest potential in single-gene barcoding for barnyard grass species identification. Cluster analysis based on *ITS* barcode sequences was performed to group the populations into five main categories. Additionally, SCoT marker analysis using six primers was performed to classify the 46 barnyard grass samples into five groups. The results showed that the predominant barnyard grass species in Ningxia were *E. colona*, *E. crus-galli var. Formosensis*, *E. crusgalli*, *E. oryzoides*, and *E. crusgalli var. Zelayensis*, with *E. colona* being the most prevalent. The differences observed between the morphological and molecular marker-based classifications were method-dependent. However, both SCoT molecular marker technology and DNA barcoding contributed to identifying the genetic diversity of barnyard grass. Taken together, our study revealed significant morphological and genetic variations among barnyard grass populations, which correlated with herbicide sensitivity in Ningxia’s paddy fields, underscoring the necessity for an integrated weed management approach to combat this troublesome weed species.

## 1. Introduction

The genus *Echinochloa*, commonly known as barnyard grass, is a particularly widespread and problematic weed species found in paddy fields across the globe [1]. It poses a significant threat to paddy production, often leading to substantial yield losses. The taxonomic classification of the *Echinochloa* genus is notably intricate, primarily due to its pronounced morphological variability. Traditionally, *E. crus-galli* has been regarded as the predominant *Echinochloa* species in Ningxia (China). However, our surveys conducted in Ningxia’s paddy fields have revealed the concurrent presence of *E. colona* and *E. oryzoide* populations alongside *E. crus-galli*, underscoring the complexity of barnyard grass taxonomy in the region. Phenotypic and genetic diversities are pivotal factors influencing weed adaptation to agroecosystems [2]. Notably, herbicide resistance has been reported to correlate with the morphological characteristics of the weed [3]. For instance, Tsuji et al. [4] tested 15 different herbicides on eight *Echinochloa phylopogon* and found that the morphological differences in plant height, blade length, and spikelet length among the populations were consistent with the level of sensitivity to different herbicides. Therefore, a comprehensive understanding of barnyard grass diversity within a specific field represents an essential prerequisite for effective weed management.

Genetic diversity encompasses the total genetic information carried by all organisms on Earth, encompassing genetic and chromosomal variations. It underlies biological diversity and reflects the genetic variability and diversity among species [5]. Morphological diversity, on the other hand, refers to the diversity manifested in organisms through their morphology, structure, physiological functions, and other characteristics [6]. It serves as the visible expression of organisms adapted to their environment. Both morphological methods and DNA techniques can aid in identifying genetic variations. However, each approach has its strengths and limitations, and no single technique can fully replace the other. Utilizing morphological traits to assess genetic variation remains a straightforward and uncomplicated approach, providing a fundamental basis for barnyard grass taxonomy. Nevertheless, changes in agricultural activities, herbicide application, and shifting climatic conditions have induced significant alterations in barnyard grass morphology [7], leading to varying perspectives among researchers regarding its classification. Consequently, establishing a standardized criterion for the taxonomic identification of *Echinochloa* remains a challenge.

The identification of molecular markers represents a research method that enables the direct analysis of nucleotide sequence variations [8]. Compared to morphological identification, this approach remains unaffected by seasonal changes, environmental factors, or the developmental stage of the plant. On the other hand, DNA barcoding serves as a rapid method for identifying and characterizing plant species using one or a few standardized DNA fragments [9]. The application of plant DNA barcoding allows for an accurate and swift identification of plants on a large scale, irrespective of individual plant morphology and growth stage. However, for DNA barcoding to be effective, there must be a sufficient degree of sequence variation between species to enable clear differentiation, while within a species, the variation must be limited to define distinct thresholds for intraspecific and interspecific genetic differences. Zhang et al. [10] used the chloroplast DNA barcode *psbA* to categorize over 200 barnyard grass specimens into four groups. Similarly, Amaguchi et al. [11] utilized the chloroplast barcode *trnT-L-F* to classify nine barnyard grass species into five groups. Lahaye et al. [12] successfully amplified 1667 plant materials using the chloroplast barcode *matK*. Taberlet et al. [13] recommended the chloroplast barcode *trnL* intron as a suitable plant barcode. Another commonly used fragment for plant DNA barcoding is the intra-ribosomal transcribed spacer region *ITS*. In the present study, we selected chloroplast DNA barcodes (*psbA*, *trnL-F*, and *matK*) and the nuclear DNA barcode (*ITS*) as candidate sequences to evaluate their discriminative capacity for *Echinochloa* spp. in Ningxia to establish standardized DNA barcode sequences applicable to *Echinochloa* spp., providing a valuable reference for molecular identification. 

Traditional random DNA molecular markers (RDMs) have demonstrated their effectiveness and reliability through extensive applications. One such targeted gene molecular marker is the Start Codon-Targeted Polymorphism (SCoT) marker, which was developed by Collard and Mackill [14]. SCoT molecular markers exploit the highly conserved and consistent sequence flanking the ATG start codon, similar to RAPD and ISSR markers. SCoT primers are designed to bind to the ATG translation initiation site on both the positive and negative DNA strands, facilitating the amplification of DNA fragments between these binding sites. SCoT markers represent valuable complements to RAPD and ISSR markers [15]. SCoT markers can be used to trace specific traits and identify trait-related target genes, providing a clearer depiction of the relationships and genetic structure among diverse resources. The technology involving SCoT gene targeting markers has found widespread application in plant genetics and genomics research, spanning various domains, including, Iraqi barley [16], medicinal plants [17], vegetables [18], trees [19,20,21], and crops [22]. However, few studies have been conducted to identify the biodiversity of barnyard plants based on SCoT molecular markers. 

In Ningxia, there are usually four to five distinct species of the *Echinochloa* genus in paddy fields. However, there is a lack of information on barnyard grass populations’ genetic and morphological diversity, and these weed populations remain poorly characterized. To address this knowledge gap, we employed a comprehensive approach integrating morphological analysis, SCoT molecular markers, and DNA barcoding technology to thoroughly assess the genetic and morphological diversity among 46 distinct barnyard grass populations in Ningxia’s paddy fields.

## 2. Results

### 2.1. Analysis of Quantitative Trait Variation and Diversity

To assess the variation and diversity in barnyard grass quantitative traits in Ningxia, we employed various statistical measures, including Range, Max., Min., mean, coefficient of variation (CV), and diversity index (H′) (Table 1). The diversity index ranged from 0.69 to 2.06, with an average of 1.69, reflecting the diversity within these traits. The coefficient of variation, which signifies how much the traits varied with their mean values, ranged from 11.69% to 221.91%, with an average of 44.01%. Then, we considered a combination of the diversity index, coefficient of variation, and the extent of variation and found that among the quantitative traits, leaf length, stem diameter, and plant height exhibited significant genetic differences.

Quantitative traits are controlled by multiple genes and are characterized by continuous variation. Moreover, the quantitative traits are normally distributed due to the influence of the genotype frequency. In this study, we divided the genotypes into 10 levels and calculated their genotype frequencies (Appendix A, Table A1). The normality of each trait was analyzed using the Shapiro–Wilk test (Appendix B, Table A2). All traits were normally distributed, except for awn length, which was generated due to the low richness of the awn length of the test samples. However, there are significant morphological differences in different populations, and considering that awn length is also an important indicator of weed morphological classification, it can be used for genetic diversity analysis.

### 2.2. Correlation Analysis of Morphophysiological Traits and Their Association with Herbicide Resistance

The clustering analysis of nine quantitative traits of 46 test materials of *Echinochloa* was performed using the SPSS 24.0 software. We constructed a dendrogram for all the test materials using the Ward method. At a genetic distance of 15.0, the 46 barnyard grass materials were categorized into five distinct clusters. Briefly, Cluster 1 was characterized by broad and long leaves, extended inflorescences, the absence of awns, lengthy spikelets, and tall and robust plants. The average plant height within this group reached 89.13 cm, with an average leaf length of 39.0 cm. Cluster 2 featured short leaves and awns, shorter spikelets, and slender stalks. The average leaf length for this cluster was only 26.59 cm. Cluster 3 exhibited slender leaves, longer awns, extended spikelets, compact plants, and sturdy stalks, with the average main stem diameter in this cluster reaching 0.66 cm and the highest being 0.81 cm. Cluster 4 had slender leaves, long awns, extended spikelets, taller plants, and slender stalks. A noteworthy feature of this group was the awn length, with an average of 0.81 cm. Cluster 5 displayed slender leaves, shorter inflorescences, the absence of awns, shorter spikelets, and diminutive, slender plants. The plants within this cluster were notably small, with an average plant height and main stem diameter of only 43.35 cm and 0.29 cm, respectively.

**Figure 1:**
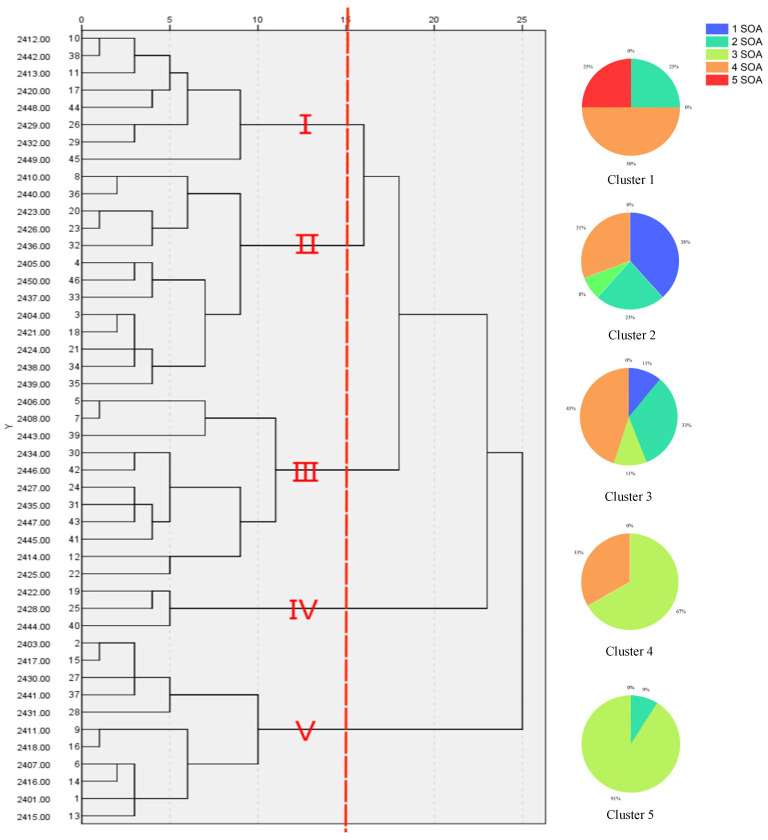
After standardizing the mean value of the quantitative indexes of various populations, a cluster analysis map was drawn using the results of the nine standardized quantitative indicators. I–V represent the five cluster groups obtained based on the results of cluster analysis. Hierarchical cluster analysis for the 46 *Echinochloa* spp. and the herbicide resistance status for the ecotypes grouped within each cluster are shown in pie charts. Colors indicate resistance to one or more herbicide sites of action (SOAs). For example, 2 SOAs represents the presence of two different sites of action of fresh weight inhibition by herbicides of less than 78%.

**Figure 2:**
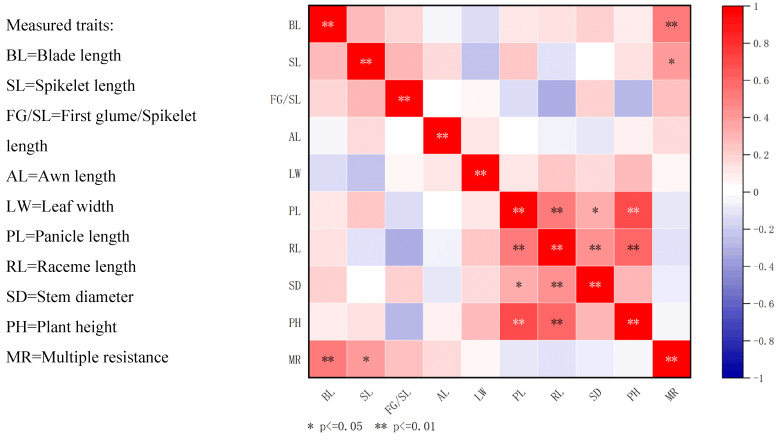
Correlation analysis between herbicide resistance status and the 8 morphophysiological traits measured in 46 *Echinochloa* ecotypes. The eight morphological traits correlated with multiple resistance are shown in the figure’s bottom row or right column. Information regarding the “Correlation between population morphology and multi-resistance” can be found in Appendix D, Table A4. ** *p* ≤ 0.01, * *p* ≤ 0.05.

In previous studies, we assessed the herbicide resistance of all samples using six different herbicides. The multiple herbicide resistance profiles of barnyard grass within each cluster are shown in Figure 1. We observed significant associations between certain phenotypic traits and herbicide resistance status (Figure 1). Specifically, leaf length and spikelet length exhibited a positive correlation with multi-resistance (Figure 2, Appendix C, Table A3). In addition, Cluster 1 displayed the most notable leaf length feature among all clusters: 25% of biotypes were resistant to all five herbicides, and 50% of biotypes demonstrated resistance to four of the herbicides. In Cluster 3, the abundance of herbicide resistance profiles was high, with not only the presence of 44.4% of the population resistant to the four herbicide sites of action (SOAs) but also the presence of 11.1% that were not multi-resistant. Cluster 4 consisted of three ecotypes, with two of them being resistant to three SOAs and one ecotype resistant to four SOAs. In contrast, Clusters 2 and 5 featured plants with shorter leaf lengths and spikelets. In Cluster 2, comprising a total of 13 ecotypes, more than half were resistant to one and two SOAs. The proportion of multi-resistance was lowest in all cluster groups. In Cluster 5, 9.1% of the biotypes exhibited resistance to two SOAs, while the remaining biotypes were resistant to three SOAs.

### 2.3. Analysis of DNA Barcoding and SCoT

#### 2.3.1. DNA Barcoding Analysis

**Figure 3:**
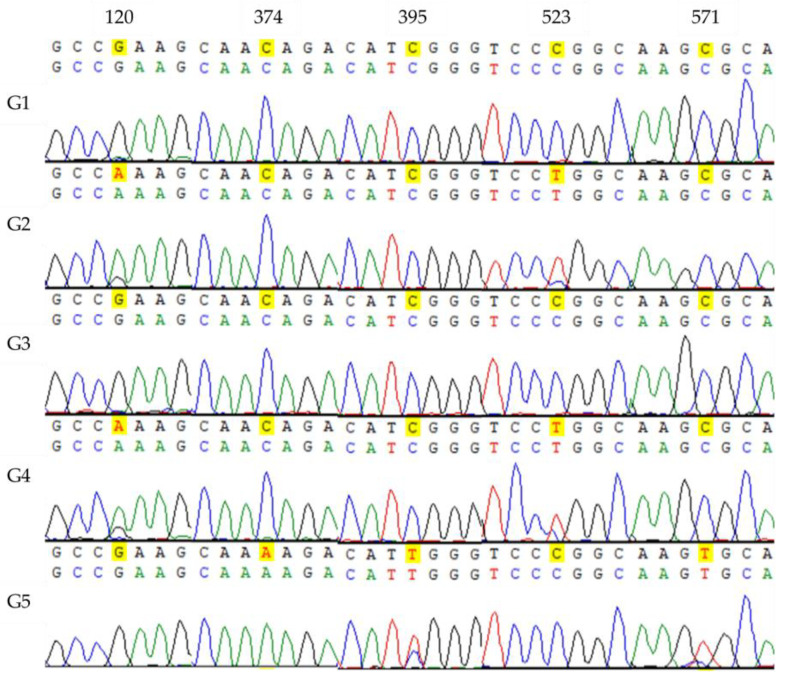
*ITS* sequence difference sites and corresponding clustering patterns. The *ITS* single-nucleotide polymorphism (SNP) sites depicted in the figure correspond to specific positions within the known *ITS* sequences of reference strain KP711096.1 (GenBank accession number). Group designations (G1–G5) are assigned to represent distinct genotypes identified through phylogenetic analysis. Each group (G1, G2, G3, G4, G5) encompasses sequences sharing unique SNP profiles, reflecting evolutionary divergence or ecological adaptation.

**Figure 4:**
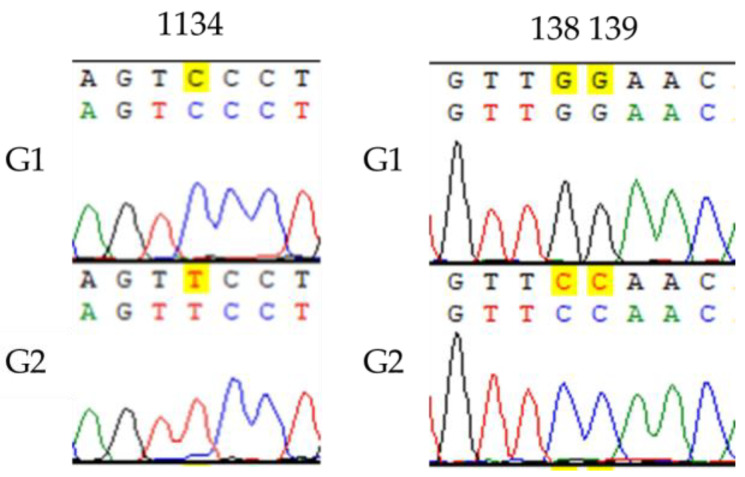
*matK* and *psbA* sequence difference sites and corresponding clustering patterns. **Left figure**: *matK*; **right figure**: *psbA*. The *psbA* and *matK* SNP sites in the figure correspond to the sites in the known sequences of HQ600076.1 and AF164422.1 in Genbank, respectively. G1 and G2 represent the different genotypes.

The sequencing results of *psbA*, *matK*, *trnL-F,* and *ITS* fragments from the 46 samples were compared by shearing, and the results showed significant differences in sequence length and the number of nucleotide differences among different barcode fragments. The amplification lengths of *psbA*, *matK*, *trnL-F* and *ITS* fragments were 337 bp, 1312 bp, 954 bp and 588 bp, respectively, and the success rate of PCR amplification and sequencing was 100%. Considering there were no nucleotide differences in the *trnL-F* fragments of the samples, the intraspecific genetic distances were all 0 and were not used for the subsequent analysis. The *psbA*, *matK* and *ITS* fragments exhibited variations at two, one, and five nucleotide positions, respectively (Figure 3, Figure 4, Appendix E, Table A5). Among these, the *ITS* sequences demonstrated the highest capacity to distinguish samples from different regions. Further analyses were conducted to explore the differential genetic diversity using *ITS* fragments.

**Figure 5:**
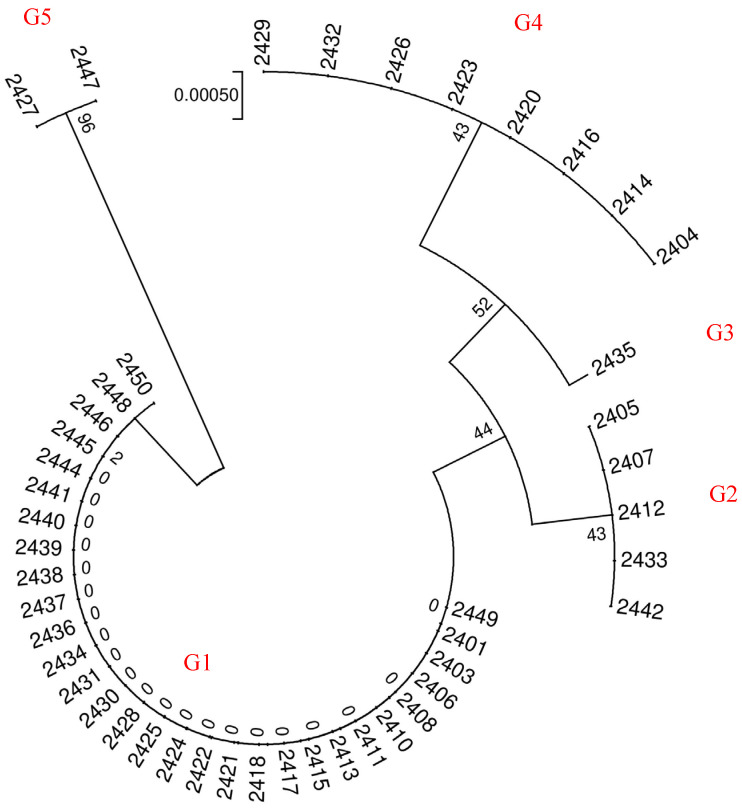
Cluster analysis of *ITS* in 46 populations. Through phylogenetic analysis utilizing *ITS* barcoding, 46 populations have been categorized into 5 distinct clusters, which are designated as group designations (G1–G5) to represent distinct genotypes.

**Figure 6:**
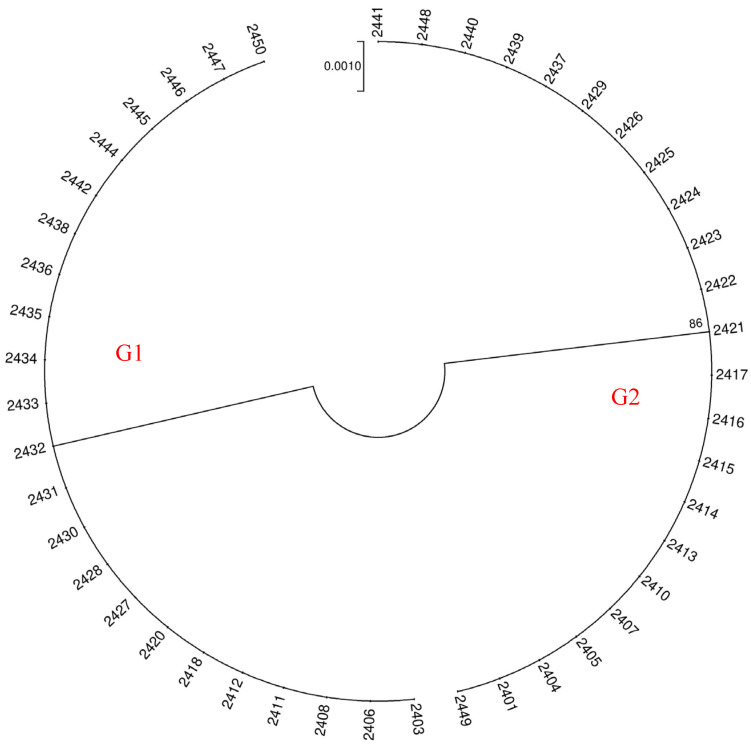
Cluster analysis of *psbA* and *matK* in 46 populations. Through phylogenetic analysis utilizing *psbA* and *matK* barcoding, 46 populations have been categorized into 2 distinct clusters, which are designated as group designations (G1 and G2) to represent distinct genotypes.

**Figure 7:**
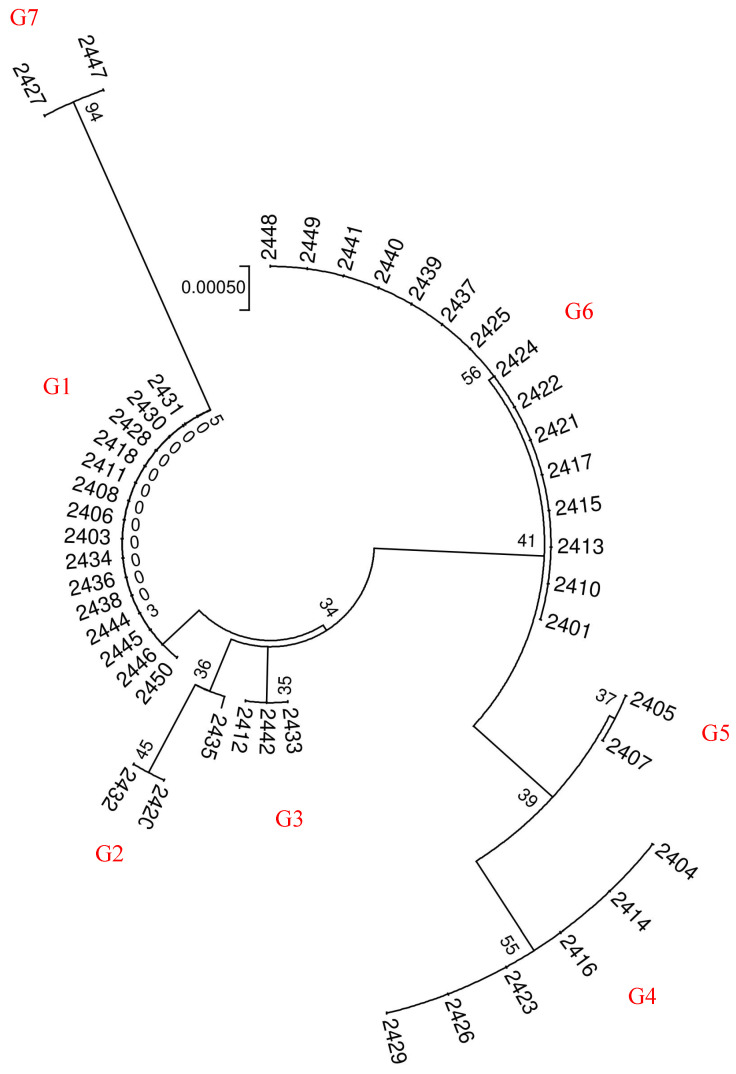
Cluster analysis of *ITS* + *psbA* in 46 populations. Through phylogenetic analysis utilizing *ITS* + *psbA* barcoding, 46 populations have been categorized into 7 distinct clusters, which are designated as group designations (G1–G7) to represent distinct genotypes.

To visually assess the discriminatory capability of different candidate barcode sequences, we constructed NJ trees for each of the three sequences. Using the *ITS* barcode, we classified the 46 barnyard grass samples into five distinct groups (Figure 5). On the other hand, both the *psbA* and *matK* barcodes could group all samples into two clusters (Figure 6). During our analysis of differences in single barcoded gene fragments among samples, we observed consistent clustering patterns for *psbA* and *matK*. However, we also noted sequence variations among samples with identical *ITS* sequences for either *psbA* or *matK*. Consequently, we combined the *ITS* and *psbA* fragments to cluster all samples, classifying the 46 barnyard grass populations into seven groups (Figure 7).

From the results of genetic variation and the construction of NJ trees in this study, we found that a single DNA barcode sequence cannot achieve 100% identification of barnyard plants. Due to the best clustering effect of *ITS* barcode sequences, *ITS* was considered the core barcode for barnyard plant identification. Additionally, we recommend the utilization of multiple barcode fragments in combination to enhance the accuracy of species identification.

According to the *Flora of China* [23], *Echinochloa oryzoides* (Arduino), Fritsch typically exhibits a dense bundle of hairs on the abaxial surface at the junction of the leaf sheath and blade. The morphology of *E. colona* is characterized by a weak plant with spikelets arranged in regular rows of four to one side of the rachis. *E. crusgalli* is an erect, stout plant with an erect panicle. *E. crus-galli* var. *mitis* is awnless and the branches on the raceme are often rebranched, and the branches on the raceme of *E. crusgalli* var. *zelayensis* are no longer branched.

Furthermore, our chromosome ploidy analysis revealed that G5 has a chromosome count 2n = 36. As a result, we can accurately identify samples 2427 and 2447 as *Echinochloa oryzoides*. Precise identifications could be achieved by comparing the sequences of groups G1-G4 within the *ITS* clustering results with sequences available in Genbank. Specifically, the G1, G2, and G4 groups were accurately matched with *Echinochloa colona* (MH808815.1), *Echinochloa crus-galli* var. *Formosensis* (LC334436.1), and *Echinochloa crusgalli* (OR678342.1), respectively. The G3 group of samples aligned with the morphological description of *Echinochloa crusgalli* var. *Zelayensisde*. This result is consistent with the morphological characteristics of each barnyard grass species.

#### 2.3.2. SCoT-PCR Analysis

**Figure A1:**
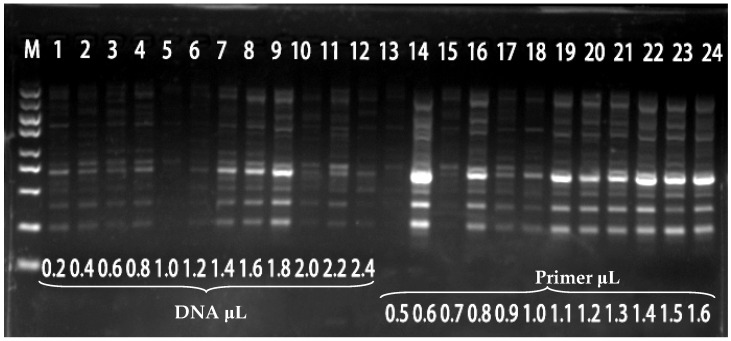
Single-factor optimization of PCR reaction system. Note: M: DL5000 DNA marker. Numbers 1~12 are the results of electrophoresis of different DNA templates. Numbers 1~12 represent the lanes of electrophoresis of different DNA template additives, and 13 to 24 are the results of different primer additions. The optimal addition amount of the two factors was selected by visual inspection.

**Figure A2:**
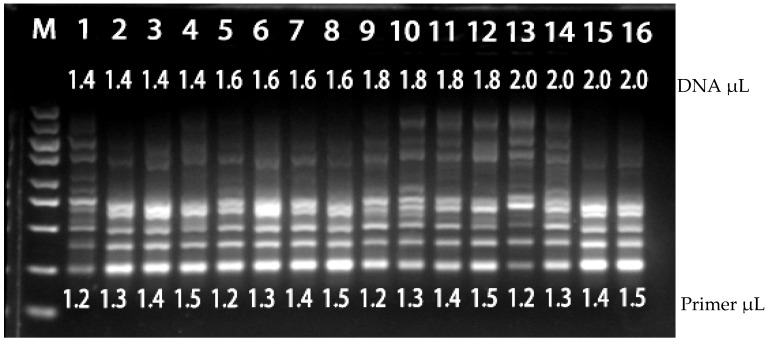
SCoT-PCR orthogonal experiment. Note: M: DL5000 DNA marker. 1–16 correspond to different orthogonal experimental groups, and the optimal experimental group is selected by observing the quantity and clarity of electrophoretic bands.

We compiled the optimization results and the analysis of orthogonal experiments of the SCoT-PCR reaction system. The results of single-factor experiments indicate that the best outcomes occurred when adding 1.4 μL to 2.0 μL of DNA template and 1.2 μL to 1.5 μL of primer (Appendix G, Figure A1). Among the 16 groups in the orthogonal experiment (Appendix H, Figure A2), the most favorable amplification results were observed in group 10, where we used 1.8 μL of DNA template and 1.3 μL of primer.

**Figure A3:**
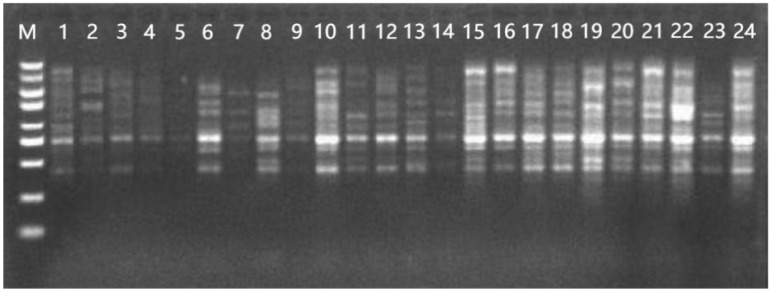
The electrophoresis results of SCoT 20 for some of the samples. Note: M: DL5000 DNA marker, 1–24 represent population 2401–2427 in Table A6, respectively. This picture only shows the SCoT 20 electrophoresis results of some populations, and all the SCoT results were verified several times in order to achieve a bright and clear polymorphic band, which is not fully shown here.

Following the single-factor experiments and orthogonal optimization, we established the SCoT-PCR system, comprising 1.8 μL of DNA, 1.3 μL of primers, 12.5 μL of 2× TIANGEN Taq Plus PCR mix, and ddH_2_O added to reach a final volume of 25 μL (Appendix I, Figure A3). Overall, we assessed six primers and applied them to amplify 46 barnyard grass DNA samples via PCR. By comparing samples from 46 populations at the same electrophoretic band size position, we defined loci where all samples exhibited the same band as non-polymorphic loci, while those showing variations were designated as polymorphic loci. The outcome revealed 72 amplified bands, with 62 of them displaying polymorphisms. The number of bands varied per primer, ranging from 9 (SCoT12) to 15 (SCoT20), averaging 12 bands per primer. Taken together, the SCoT primers produced a total of 72 band loci, of which 62 were polymorphic. On average, each primer generated 10.3 polymorphic loci, resulting in an average polymorphism rate of 86.1%. Particularly, the SCoT20 primer exhibited the highest level of polymorphism, at 93.3%. Among the six SCoT primers, the *PIC* (polymorphism information content) coefficients ranged from 0.25 (SCoT11) to 0.39 (SCoT12) (Table 2). The relationship between *PIC* coefficients and SCoT primers indicates that different primers vary in their ability to detect genetic variation. SCoT12 can more effectively reveal the genetic diversity of barnyard grass.

**Figure 8:**
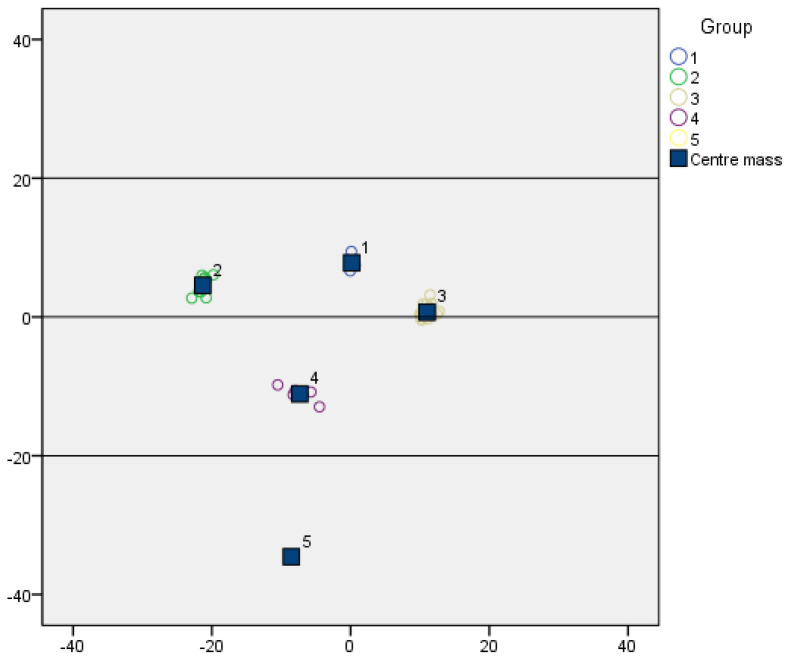
Bayesian analysis-based genetic similarity assay. The plot demonstrates the grouping and center of mass of genetic samples based on their similarity. Each point represents a genetic sample, and the numbered points (1–5) indicate distinct groups identified through clustering. The dark blue squares represent the center of mass for each group, providing a visual representation of the genetic similarity and grouping patterns among the samples.

In this study, we conducted a clustering analysis of genetic samples using the SCoT (start codon targeted) molecular marker technique. Through Bayesian analysis, we successfully categorized the samples into five distinct groups (labeled 1 to 5). Each group exhibited significant differences in genetic similarity, which were intuitively demonstrated by their relative positions in the diagram (Figure 8).

The clustering results revealed that Groups 1 and 2 are genetically closer to each other, with their centroids (dark blue squares) positioned near each other in the diagram, indicating a high degree of similarity between these two groups of samples in terms of SCoT molecular markers. In contrast, Group 5 displayed substantial genetic differences from the other groups, with its centroid located far from those of the other groups in the diagram, showcasing unique genetic characteristics.

Although Groups 3 and 4 also formed independent clusters, their genetic distance was relatively close, suggesting a certain level of similarity between these two groups of samples in terms of SCoT molecular markers, yet they were still distinct from the other groups.

**Figure 9:**
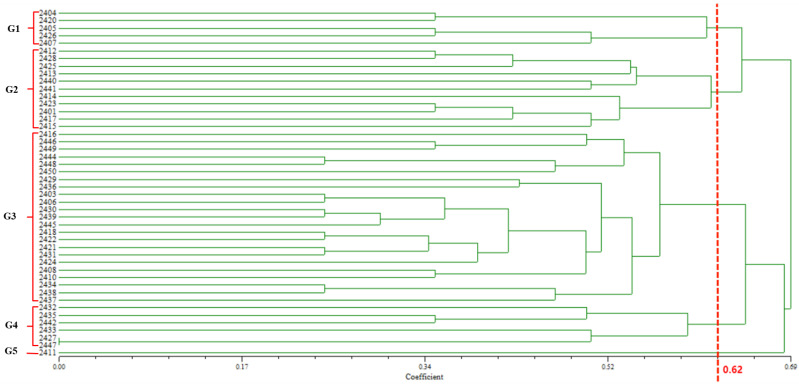
Cluster analysis of SCoT molecular markers in 46 populations. In total, 62 polymorphic genes were used by 46 populations to conduct a 0/1 scoring, upon which a cluster analysis was performed. The red dashed line indicates the threshold position for cluster classification, which is at 0.62.

We constructed a cluster analysis graph utilizing the specificity matrix. The coefficients of variation among the 46 barnyard grass samples ranged from 0.00 to 0.69 (Figure 9). Most samples exhibited coefficients of variation between 0.24 and 0.69, indicating substantial differences among the samples collected in this experiment. With a genetic coefficient of variation set at 0.62, the 46 barnyard grass samples were divided into five groups, aligning with the clustering observed in the *ITS* DNA barcodes, potentially due to variations in intraspecific identification among different molecular markers. Notably, SCoT molecular markers exhibited a higher discriminatory capacity in the species.

### 2.4. Correlation of the Different Detection Means

The correlation of the different identification means was analyzed by standardizing the scores of the cluster analysis results. The data in Table 3 indicates that the correlation index between *ITS* markers and SCoT molecular markers was 0.76, which is significant. It also indicates that the results of the two molecular markers have consistency. There is also a significant correlation between *ITS* and morphological trait clustering in molecular marker technology, but SCoT is different. Genes marked by SCoT are not identical to genes regulating morphological traits, so the correlation in the cluster analysis results is low. This result also shows gene diversity within phenotypically consistent species. We have assigned values from 1 to 5 to populations exhibiting consistent *ITS* single-nucleotide polymorphisms among the 46 populations. Similarly, SCoT markers were assigned based on the coefficient of variation (0.62), as illustrated in Figure 9, and were separately allocated according to cluster analysis. Morphological markers were also assigned in accordance with cluster analysis results. Subsequently, correlation and significance analyses were conducted on these three assignments. A significant correlation is observed between *ITS* and SCoT markers, and there is also a notable correlation between *ITS* and MT markers.

## 3. Discussion

Genetic diversity plays an important role in overall biodiversity. Species with greater genetic diversity are better equipped to adapt to changing environmental conditions [24]. As observed in prior studies, our research confirmed the extensive variability among barnyard grass species based on numerous morphological and biological characteristics. These findings are consistent with the outcomes of a genetic diversity analysis of barnyard grass by Lu [25]. Furthermore, we identified a positive correlation between certain phenotypic traits and the number of herbicide resistance sites among the tested biotypes. Herbicides play a role in driving morphological variations in weeds and favoring the dominance of specific populations.

DNA barcoding relies on standardized short genomic regions with sufficient sequence variation to distinguish between species [26,27]. In this study, *ITS* demonstrated superior distinctiveness compared to other sequences, making it more suitable for barnyard plant identification. In our experiment, the combination of *ITS* and *psbA* barcodes provided enhanced discrimination of *Echinochloa* spp. The recognition ability of DNA barcodes is related to the number of combinations [28]. SCoT marker technology, effective in generating trait-linked genetic markers, shows significant value in genetics. To establish a stable, reproducible system, we used orthogonal design to optimize these key variables, successfully constructing a robust SCoT reaction system. Therefore, in this study, we used six SCoT primers to analyze 46 barnyard grass populations, resulting in a high percentage of polymorphic loci (86.1%), thereby highlighting the effectiveness of SCoT markers in assessing barnyard grass biodiversity.

When comparing genetic diversity assessed by molecular markers with the results obtained from morphological analysis, we often observe discrepancies between the two. The fundamental cause of the discrepancies observed in cluster analysis lies in their inherently distinct genetic information bases [29]. This is because genetic similarity primarily hinges on relatively stable genomic information, as supported by the research conducted by Hamrick and Liu [3,30]. Molecular marker technology detects variations at the DNA sequence level of organisms, encompassing base substitutions, insertions, or deletions within the genetic material [31]. These variations are marked by high stability and heritability, remaining unaffected by the organism’s developmental stage, environmental conditions, or phenotypic complexity. For example, molecular marker technology can precisely elucidate genetic disparities and kinship among geographically distinct populations of the same species, regardless of their climatic adaptations. Molecular marker technology can scan the entire genome and detect numerous genetic variation loci, offering a more comprehensive view of an organism’s genetic diversity [32]. Many traits in organisms are jointly controlled by multiple genes with complex interrelationships [33]. Molecular marker technology, through methods such as association analysis, can identify multiple gene loci associated with specific traits and analyze their interaction patterns, providing a robust tool for understanding the genetic mechanisms of organisms.

In contrast, the manifestation of morphological traits arises from the interplay between gene expression and environmental conditions, with even minor environmental changes potentially inducing substantial alterations in these traits. For instance, in this study, the leaf length (M1) and plant height (M9) of barnyard grass were identified as two pivotal morphological traits that are significantly influenced by environmental factors, thereby demonstrating notable adaptive variations. Specifically, within the Yinchuan region, which is characterized by favorable irrigation practices and abundant soil moisture (e.g., sampling sites ID 2401, 2404), barnyard grass exhibited elongated leaves and increased plant stature. The data revealed that the mean leaf length in these areas surpassed 80 cm. Conversely, in arid environments, such as the Shapotou District in Zhongwei City (e.g., sampling sites ID 2421, 2422), barnyard grass adapted to the harsh conditions by reducing leaf length and plant height. Quantitative data indicated that the average leaf length in these regions fell below 60 cm. These morphological alterations constitute an adaptive strategy employed by plants to mitigate drought stress, where reduced leaf area minimizes water loss and decreased plant height mitigates the effects of wind and soil erosion, ultimately enhancing their prospects for survival under drought conditions.

In contrast, morphological trait measurement typically only reflects the effects of a few key genes or gene combinations. While the formation of morphological traits is influenced by multiple genes, in practice, we often select only a few easily observable and measurable morphological traits for analysis. These traits may only represent the expression effects of a subset of genes, limiting the ability to fully reveal an organism’s genetic complexity. Additionally, morphological traits can be affected by epigenetic modifications [34], such as epigenetic marks that alter gene expression patterns without changing the DNA sequence, further complicating the use of morphological traits in reflecting an organism’s genetic essence.

Phenotypic differentiation in *Echinochloa* spp. is shaped by the interplay of genotype, environment, and their interactions, exhibiting a hierarchical adaptive mechanism. Phenotypic plasticity serves as the primary mediator of environmental responses [35]: under high-water conditions in Yinchuan (e.g., Helan County), barnyard grass elongates leaves (M1 = 104.40 cm) and increases plant height to enhance photosynthetic efficiency. Conversely, in drought-stressed Zhongwei (e.g., Shapotou District), populations shorten leaves and thicken stems (M8) to reduce transpiration, with significant height differences observed between samples 2411 (64.62 cm) and 2415 (104.40 cm), confirming environment-driven phenotypic divergence. Soil nutrient gradients further amplify morphological variation; for instance, nitrogen enrichment in Shizuishan (Pingluo County) promotes tillering (M3) and spikelet length (M4), while nutrient-poor soils in Wuzhong (Litong District) inhibit morphological development.

Herbicide selection pressure and microenvironmental stressors accelerate adaptive morphological evolution: prolonged exposure to 34% propanil in populations (e.g., Cluster 1) enhances metabolic detoxification via increased leaf length (M1 = 39.0 cm) and biomass accumulation, supporting the hypothesis of a positive correlation between leaf length and herbicide resistance. In contrast, windblown sand in Shapotou District induces stem thickening (M8) without genetic variation detected by *ITS* markers, indicating that morphological changes are primarily stress-induced.

Geographic isolation and restricted gene flow accelerate population differentiation, yet phenotypic plasticity may obscure genetic differences [36]. For example, *ITS*-based genetic divergence exists between Yinchuan and Lingwu populations separated by the Helan Mountains, despite morphological convergence due to similar irrigation practices. In contrast, Zhongwei’s arid populations have shortened awns (M5) to adapt to drought while retaining genetic diversity in SCoT markers, reflecting the decoupling of morphological and genetic adaptation.

Genotype-by-environment interactions (G×E) result in context-dependent phenotypic expression within the same genetic background. In Wuzhong, diurnal temperature fluctuations induce variation in tillering (M4) and spikelet morphology, yet SCoT markers remain genetically similar to Yinchuan populations, suggesting that phenotypic changes are environmentally driven. Intensive agricultural management in Yinchuan reduces plant height (e.g., Cluster 5, 43.35 cm) without detectable genetic differentiation in *ITS* analysis, indicating that anthropogenic disturbances accelerate adaptive evolution through phenotypic selection, with genetic divergence lagging behind.

The divergences between morphological and molecular classification reflect the adaptive strategies of *Echinochloa* species in complex environments. The combined effects of geographic isolation, herbicide selection pressure, and phenotypic plasticity have led to a decoupling between phenotypic traits and genetic backgrounds. In the future, it is essential to integrate environmental data with multi-omics technologies to comprehensively elucidate the evolutionary dynamics of *Echinochloa*, thereby providing a theoretical basis for precise prevention and control measures.

## 4. Materials and Methods

### 4.1. Plant Materials

**Figure 10:**
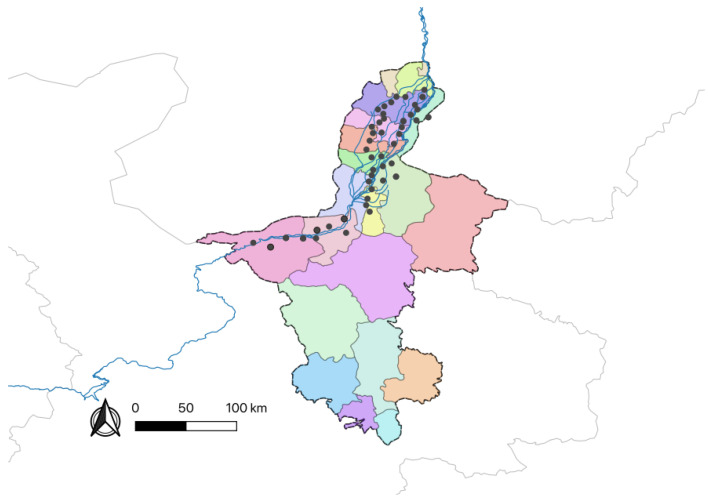
Distribution map of seed collection sites for *Echinochloa* spp. in Ningxia region.

Barnyard grass seeds were collected from rice cultivation areas across four regions in Ningxia province, Yinchuan, Zhongwei, Wuzhong, and Shizuishan, during September 2021. Seeds displaying a uniform morphology and maturity harvested from the same field were categorized into a single population group. Within each sampling site, the barnyard grass species remained consistent, representing dominant populations that had adapted to and withstood long-term environmental pressures. Seeds from the same sampling site were pooled and stored separately, with a minimum 10 km separation distance or distinct geographical barriers such as rivers and mountains between collection sites to minimize potential genetic mixing. A total of 46 barnyard grass populations were collected across the study region (Figure 10, Table A6), with detailed collection information provided in Appendix A. Mature barnyard grass seeds were placed in labeled nylon mesh bags, dried in the sun, and subsequently stored at room temperature for use.

Seeds from all 46 barnyard grass populations were soaked in water for 24 h, followed by surface drying on filter paper. For each population, ten uniformly mature seeds were sown in 14 cm diameter plastic pots filled with potting mix comprising 1:1 (*v*/*v*) peat and sand. Seeds were covered with approximately 0.5 cm layer of soil to optimize germination conditions while accommodating complete life cycle development. The pots were cultivated in the greenhouse of the Institute of plant Protection, Chinese Academy of Agricultural Sciences, China. Sampling was conducted until the plants reached the 2–3 leaf stage of growth. We collected leaf tissue samples from 10 samples per population for DNA extraction and stored at −20 °C for subsequent analysis. After sampling, the remaining plants were carefully transplanted into individual 14 cm pots containing growth substrate. Plants were maintained in a green house until full maturity, at which point the morphological indexes of the plants were assessed.

Sampling locations (*n* = 46) are marked with black dots corresponding to different populations.

### 4.2. Multi-Resistance Assay

Barnyard grass samples cultured in 4.1 were used to determine susceptibility to six different herbicides at the three-leaf stage using whole-plant experimental assays. The six herbicides and the maximum recommended field dosage were 10% metamifop 120 g a.i/hm^2^, 25% penoxsulam 300 g a.i/hm^2^, 10% pyribenzoxim 45 g a.i/hm^2^, 34% propani 4498.2 g a.i/hm^2^, 25% quinclorac 300 g a.i./hm^2^, and 40% cyhalofop-butyl 120 g a.i/hm^2^, respectively. Each herbicide was treated using a 3WP-2000 type self-propelled spray tower (manufactured by Nanjing Institute of Agricultural Mechanization, Nanjing, China) with a flat–fat nozzle under the following operational conditions: a travel distance of 1320 mm and a constant speed of 412 mm/s. The cultivation conditions are set as L/D = 13 h, (30 ± 5) °C // 11 h, (25 ± 5) °C. Fourteen days after herbicide application, the aboveground fresh weight of each barnyard grass sample was weighed. The fresh weight suppression rate of different barnyard grass populations was then calculated to untreated controls. The biotypes with a fresh weight inhibition rate lower than 78% were defined as resistant biotypes. Three replicate pots containing 10 healthy and vigorous plants per pot were used, and the entire experiment was conducted twice.

### 4.3. Morphological Traits

The morphological characteristics of 46 barnyard grass samples were observed and described in accordance with taxonomic standards from the *Flora of China* and the morphological framework established by Zou Manyu et al. [37]. Nine quantitative traits were measured at the reproductive growth stage, denoted as M1 to M9: (M1) leaf length, (M2) leaf width, (M3) panicle length, (M4) raceme length, (M5) awn length, (M6) spikelet length, (M7) first glume length relative to spikelet length, (M8) main stem diameter, and (M9) plant height (Table 4). For each population, 10 healthy-growing plants are selected for measurement, with the mean value calculated for analysis.

### 4.4. DNA Extraction

To prepare the samples, 100 mg of tender apical leaves tissue was collected and transferred to individual 1.5 mL centrifuge tubes. Additionally, we added two sterile steel beads, each with a diameter of 3 mm, into each tube. Samples were immediately frozen in liquid nitrogen for 2 min and transformed into a fine powder using a TissueLyser II sample crusher (Beijing Tiangen Biochemical Technology Co., Ltd., Beijing, China) at a frequency of 30 Hz for 45 s. Genomic DNA extraction was performed following the protocol outlined in the Tiangen New Plant Genomic DNA Extraction Kit (DP320-03, Tiangen Biochemical Technology Co., Ltd., Beijing, China). To ensure the integrity of the DNA, we conducted a 1% agarose gel electrophoresis analysis of the samples and assessed the concentration and purity of the extracted DNA using an ultraviolet spectrophotometer (Gene Co., Ltd., Beijing, China). The DNA was then diluted to a working concentration of 50 ng/μL and safely stored in a −20 °C refrigerator for future use.

### 4.5. PCR Amplification of DNA Barcode Primers

In this study, the DNA barcoding genes of 10 samples from each of the 46 barnyard grass (*Echinochloa* spp.) populations were successfully amplified. In a 25 μL PCR reaction system, 1 μL (50 ng/μL) genomic DNA, 1 μL (10 μM) each of forward and reverse primers (for primer details, see the Table 5 provided in Gao and Yuan [38,39]), 12.5 μL 2× TIANGEN Taq Plus PCR mix, and 9.5 μL ddH_2_O were used. After 10-s centrifugation for separation, the PCR process was continued with an initial denaturation at 94°C for 3 min, followed by 34 cycles of denaturation at 94°C for 30 s, annealing at the optimal temperature for 30 s, extension at 72°C for 40–60 s, and a final extension step at 72°C for 5 min. Subsequently, the PCR products were assessed using 1% agarose gel electrophoresis, and the outcomes were analyzed with a gel image analysis system. Clear and singular bands in the PCR products were selected for subsequent bidirectional sequencing, which was performed by Beijing Biomad Gene Technology Co., Ltd., Beijing, China

### 4.6. SCoT Molecular Marker Primer Screening and PCR Amplification

#### 4.6.1. Primer Selection

The primers used for SCoT molecular markers were screened by referring to 36 SCoT primers published by Collard and Mackill [14], from which SCoT primers with strong polymorphisms and clear bands were selected.

#### 4.6.2. Single-Factor Optimization of PCR Reaction System

The 2× TIANGEN Taq Plus PCR mix was kept constant as a fixed component of the PCR system. However, we conducted univariate optimization for other variables within the system, including the quantity of barnyard grass DNA template, the addition of SCoT primers, and annealing temperatures. To ensure the reliability of our results and minimize the impact of operational errors, the experiment was replicated twice.

The six selected primers, SCoT6, SCoT11, SCoT12, SCoT20, SCoT29, and SCoT31, were configured to a concentration of 10 μM for PCR optimization (Table 6). The 12 single-factor variables for barnyard grass DNA template addition (50 ng/μL) were 0.2 μL–2.4 μL; the 12 single-factor variables for SCoT primer addition (10 μM) were 0.5 μL–1.6 μL. For each primer pair, eight temperature gradients were set up to investigate the optimal annealing temperature of each primer at Tm ± 4 °C. PCR products were detected using 1.5% agarose gel, and the results were observed using a gel imaging analysis system.

#### 4.6.3. PCR Orthogonal Optimization

We implemented a two-factor, four-level orthogonal experimental design Table 7, specifically denoted as L_16_ (4^2^), encompassing a total of 16 unique experimental groups, each repeated twice. Our analysis indicates that the evaluation of experimental results extends beyond a mere enumeration of bands. Factors such as band clarity and background characteristics play a crucial role in our assessment. Our evaluation relied on direct observation to discern the strengths and weaknesses of the treatment condition settings.

### 4.7. Data Analysis

#### 4.7.1. Morphological Trait Diversity and Cluster Analysis

Data processing and analysis were conducted using Excel 2019 to evaluate the degree of variation and diversity across each trait. Morphological diversity was quantified using the Shannon–Wiener Diversity Index (H′), calculated as follows:

H′ = − ∑PilnPi

Here, Pi represents the frequency of occurrence of the i-th variant type. Initially, the mean values of quantitative traits of 46 populations were divided into 10 levels, 1 level < X − 2 s, and 10 levels ≥ X + 2 s, with an interval of 0.5 s between each level, where X represents the mean and s signifies the standard deviation [40]. The distribution frequency of the ten levels in each trait was calculated separately.

Nine quantitative indexes of the 46 barnyard grass populations were standardized separately, and the results of the standardized quantitative indicators were used for cluster analysis. Ward’s method was employed for clustering, with Euclidean distance used as the measure of dissimilarity between germplasms. Based on the results of cluster analysis, a one-way analysis of variance (ANOVA) was conducted using SPSS 24 to statistically test the significance of differences among different geographical groups. The Student–Newman–Keuls (S-N-K) method was selected for the post hoc analysis.

#### 4.7.2. DNA Barcoding Cluster Analysis

Sequencing peaks from each sample were subjected to quality analysis using Vector NTI 11.5 software. Low-mass sequence regions were excluded, and the sequences were aligned, checked, and proofread base by base. Subsequently, multiple-sequence alignment was conducted using MEGA 6.0 software. To obtain information on DNA barcode sequence variation, the Kimura 2-Parameter (K2P) genetic distance was computed between samples. Using the K2P distance model, we constructed a phylogenetic tree through the Neighbor-Joining (NJ) method to analyze the genetic relationships among the samples. Furthermore, the Bootstrap confidence value for each branch was tested with 1000 replications to enhance the reliability of our findings.

#### 4.7.3. SCoT Molecular Marker Cluster Analysis

After the SCoT-PCR amplification, we conducted gel electrophoresis and captured images of the resulting band patterns. We then counted the distinct and clearly visible bands and scored them for each SCoT primer. These polymorphic bands served as reference points for individual random SCoT primers. Bands sharing the same position during migration were considered similar. We focused on selecting bands with well-defined and prominent backgrounds for subsequent statistical analysis, which were carefully analyzed with the Gel-Pro Analyzer 3.0 band analysis software. A control marker was introduced to simplify the analysis, which allowed us to assign values to specific migration positions based on the presence (assigned as “1”) or absence (assigned as “0”) of a band. This process yielded the initial “0/1” matrix, which formed the basis for subsequent analyses and comparisons.

The “0/1” matrix was reformatted to meet the input specifications of NTSYSpc 2.10e. To ensure data compatibility and facilitate subsequent analyses, the dataset was systematically structured and compiled into an Excel spreadsheet. The processed spreadsheet was imported into NTSYSpc 2.10e, where the Simple Matching (SM) similarity coefficient was employed to quantify pairwise genetic resemblances. The SM coefficient compares binary (“0/1”) profiles across samples, generating a genetic similarity matrix that encapsulates all pairwise genetic relationships within the dataset.

The Polymorphism Information Content (*PIC*) is used to quantify the polymorphic value of SCoT marker loci. The calculation formula is as follows:

$$PIC=1-\sum_{i=1}^{n}{\left(p_{i}\right)}^{2}$$

*n:* total number of alleles. *p_i_*: frequency of the *i*-th allele, satisfying the condition.

Prior to clustering, phenotypic data were standardized to mitigate confounding effects due to measurement scale disparities. Euclidean distances were computed based on the standardized phenotypic values, providing a quantitative metric of inter-sample dissimilarity. This distance matrix served as the basis for hierarchical agglomerative clustering (HAC) analysis.

The genetic similarity matrix and the Euclidean distance matrix were integrated to perform comprehensive clustering and systematic analysis, yielding a hierarchical clustering dendrogram that visually represents the genetic and phenotypic relationships among samples.

## 5. Conclusions

Due to the widespread adaptability of weeds, the study of their genetic mechanisms holds significant value for weed control and crop breeding [41]. A notable characteristic associated with weeds’ environmental adaptation is herbicide resistance [42,43]. Through the comparison of genetic variations between herbicide-sensitive and herbicide-resistant individuals within the same species and across different species, we can glimpse new avenues for innovating herbicide resistance mechanisms. Therefore, this study on the genetic diversity of *Echinochloa* spp. in this region contributes to our understanding of their evolutionary strategies under different ecological conditions, thereby providing a scientific basis for formulating more precise and effective weed management strategies.

## Figures and Tables

**Table 1:** Analysis of quantitative trait variation and diversity ^A^.

Traits	Range	Max	Min	Mean	S	CV (%)	H′
M1	29.45	47.50	18.05	32.59	6.81	20.91	2.02
M2	1.40	1.78	0.38	1.06	0.26	24.37	1.79
M3	7.25	13.35	6.10	9.42	1.83	19.40	2.06
M4	2.00	4.00	2.00	2.89	0.56	19.29	1.96
M5	0.96	0.96	0.00	0.10	0.21	221.91	0.86
M6	0.18	0.44	0.26	0.35	0.04	11.69	1.84
M7	0.17	0.50	0.33	0.42	0.08	20.00	0.69
M8	0.62	0.81	0.19	0.43	0.14	33.21	2.03
M9	65.43	104.40	38.97	64.62	16.34	25.29	1.99

^A^ The trait distribution indicators in the table are calculated based on the mean value of the test samples within the population. (M1) leaf length, (M2) leaf width, (M3) panicle length, (M4) raceme length, (M5) awn length, (M6) spikelet length, (M7) first glume length relative to spikelet length, (M8) main stem diameter, and (M9) plant height.

**Table 2:** SCoT primer results.

SCoT Primer	Tm (°C)	No. of Polymorphisms	Average Allele Frequency	*PIC*
SCoT06	51.70	11.00	0.64	0.35
SCoT11	58.00	12.00	0.82	0.25
SCoT12	58.80	7.00	0.69	0.39
SCoT20	64.50	14.00	0.67	0.35
SCoT29	65.70	10.00	0.72	0.35
SCoT31	63.70	8.00	0.51	0.37
Mean		10.33	0.68	0.34
Total		62.00		

SCoT—start codon targeted; *PIC*—polymorphism information content.

**Table 3:** Correlation analysis between morphology, DNA barcodes, and SCoT molecular markers clusters.

Markers		ITS	SCoT	MT
		Pearson Correlation		
ITS	*p*-value	\	0.76 **	0.36 *
SCoT		0	\	0.22
MT		0.014	0.139	\

“MT” means “Morphological Traits”; ** *p* ≤ 0.01, * *p* ≤ 0.05. The upper right triangle area of Table 3 represents the Pearson correlation of pairwise markers, while the lower left triangle area shows the significance coefficient of correlation.

**Table 4:** Assignment of 9 morphological traits of barnyard grass.

Numbers	Traits	Measuring Methods
M1	blade length (cm)	Take the third mature blade from the top of inflorescence rachis and measure its length with a ruler.
M2	blade width (cm)	Take the third mature blade from the top of inflorescence rachis and measure its width with a ruler.
M3	Panicle length (cm)	Measure the mature panicles’ length with a ruler.
M4	Racemes length (cm)	Measure the mature racemes’ length with a ruler.
M5	Awn length (cm)	Measure the awn length of the spikelets with a vernier caliper.
M6	Spikelet length (cm)	Take random mature spikelets at the top of plants and measure the spikelet length from bottom to top with a vernier caliper.
M7	The first glume/Spikelet length	Firstly, measure the first glume length with a vernier caliper and calculate the ratio of the first glume length to the spikelet length.
M8	Stem diameter (cm)	Measure the diameter of the penultimate section of the ear setting steam with a vernier caliper.
M9	Height (cm)	Measure the vertical height from the bottom to the natural top of the plant with a ruler.

**Table 5:** Primer information for barcode fragment of *Echinochloa* spp.

Barcode Fragments	Primer Name	Sequence (5′-3′)	Tm/°C	Extension Time/s
*psbA*	psb-F ^1^	GTGCCTACTCGGCATTTCAC	58.1	40
	psb-R ^2^	GTTGATAGCCAAGGTCGCGT		
*matK*	*matK*-F ^1^	TAATTTACGATCAATTCATTC	50.0	60
	*matK*-R ^2^	ACAAGAAAGTCGAAGTAT		
*trnL-F*	trn-F ^1^	ATTTGAACTGGTGACACGAG	56.3	50
	trn-C ^2^	CGAAATCGGTAGACGCTACG		
*ITS*	ITS4 ^1^	TCCTCCGCTTATTGATATGC	52.3	50
	*ITS*-Y5 ^2^	TAGAGGAAGGAGAAGTCGTAACAA		

^1^ is the forward primer, ^2^ is the reverse primer, Tm is the melting temperature.

**Table 6:** SCoT primer sequences.

Primers	Primer Sequences (5′-3′)	%GC
SCoT6	CAACAATGGCTACCACGC	56
SCoT11	AAGCAATGGCTACCACCA	50
SCoT12	ACGACATGGCGACCAACG	61
SCoT20	ACCATGGCTACCACCGCG	67
SCoT29	CCATGGCTACCACCGGCC	72
SCoT31	CCATGGCTACCACCGCCT	67

**Table 7:** L_16_ (4^2^) orthogonal experimental design.

Number	1	2	3	4	5	6	7	8	9	10	11	12	13	14	15	16
DNA	1	1	1	1	2	2	2	2	3	3	3	3	4	4	4	4
Primer	1	2	3	4	1	2	3	4	1	2	3	4	1	2	3	4

The volume of DNA added ranges from 1.4 to 2.0 μL, corresponding to DNA 1–4, respectively, while the volume of primer added ranges from 1.2 to 1.5 μL, corresponding to primer 1–4, respectively.

## Data Availability

Data are contained within the article.

## Appendix A

**Table A1:** The frequency distributions of morphological traits.

Traits/Ranks	1	2	3	4	5	6	7	8	9	10
M1	0.02	0.02	0.08	0.24	0.18	0.18	0.12	0.08	0.02	0.06
M2	0.04	0.06	0.02	0.12	0.24	0.18	0.24	0.06	0.00	0.04
M3	0.00	0.06	0.12	0.18	0.16	0.14	0.14	0.14	0.04	0.02
M4	0.00	0.06	0.10	0.26	0.08	0.20	0.12	0.10	0.08	0.00
M5	0.00	0.00	0.00	0.00	0.78	0.02	0.06	0.06	0.02	0.06
M6	0.02	0.06	0.06	0.08	0.32	0.10	0.22	0.08	0.00	0.06
M7	0.00	0.00	0.00	0.52	0.00	0.48	0.00	0.00	0.00	0.00
M8	0.00	0.02	0.12	0.26	0.24	0.06	0.12	0.12	0.08	0.04
M9	0.00	0.02	0.22	0.12	0.18	0.06	0.20	0.20	0.04	0.02

The mean distribution of quantitative traits within each group was divided into 10 ranks to calculate the frequency distribution of each rank. The frequency distributions in the table were all calculated from the mean of each quantitative trait across the 46 populations. The information on the morphological traits denoted by M1–M9 is presented in Table 4.

## Appendix B

**Table A2:** Detection of normality of morphological traits.

Normality Test (Shapiro–Wilk Test)			
Traits	Statistics	DOF	*p*
M1	0.955	46	0.07
M2	0.962	46	0.142
M3	0.977	46	0.475
M4	0.955	46	0.07
M5	0.547	46	0 *
M6	0.974	46	0.402
M7	0.637	46	0 *
M8	0.962	46	0.144
M9	0.961	46	0.122

In this study, the significance of the 46 populations was calculated using Shapiro-Wilk test assuming that “each morphological trait is not normally distributed”, * mean *p* ≤ 0.05 and is not normally distributed. The data in the table were calculated from the distribution frequency of each quantitative trait. The information on the morphological traits denoted by M1–M9 is presented in Table 4.

## Appendix C

**Table A3:** Analysis of molecular variance (AMOVA) showing morphological diversity.

Traits	SS	df	MS	F	*p*-Value
M1	732.767	4	183.192	5.089	0.002
	1403.826	39	35.996		
	2136.593	43			
M2	0.576	4	0.144	2.535	0.055
	2.217	39	0.057		
	2.793	43			
M3	79.832	4	19.958	11.346	0.001
	68.601	39	1.759		
	148.432	43			
M4	8.775	4	2.194	20.214	0.001
	4.233	39	0.109		
	13.008	43			
M5	1.647	4	0.412	27.616	0.001
	0.582	39	0.015		
	2.229	43			
M6	0.012	4	0.003	1.779	0.153
	0.063	39	0.002		
	0.075	43			
M7	0.07	4	0.017	2.735	0.042
	0.248	39	0.006		
	0.318	43			
M8	0.539	4	0.135	15.278	0.001
	0.344	39	0.009		
	0.884	43			
M9	8844.138	4	2211.034	25.68	0.001
	3357.833	39	86.098		
	12,201.971	43			

SS—sum of squares, df—degree of freedom, MS—mean of squares, F—F-value. The information on the morphological traits denoted by M1–M9 is presented in Table 4.

## Appendix D

**Table A4:** Correlation between population morphology and multi-resistance.

Traits	Case Number	Pearson Correlation	*p*
M1	46	0.53 **	0.001
M2	46	0.06	0.734
M3	46	−0.10	0.571
M4	46	−0.12	0.503
M5	46	0.17	0.331
M6	46	0.40 *	0.021
M7	46	0.28	0.118
M8	46	−0.08	0.648
M9	46	−0.04	0.825

** *p* ≤ 0.01, * *p* ≤ 0.05. The data in the table are the average values of the quantitative traits of various groups and their SOA numbers for Pearson Correlation and Significance analysis. blade length (M1), Spikelet length (M6) and multi-resistance were significantly associated. The information on the morphological traits denoted by M1–M9 is presented in Table 4.

## Appendix E

**Table A5:** DNA differential loci ^B^ with different barcodes.

Number	Populations	*ITS*					*psbA*		*matK*
		120	374	395	523	571	138	139	1134
1	2401	G	C	C	C	C	G	G	T
2	2403	G	C	C	C	C	C	C	C
3	2404	A	C	C	T	C	G	G	T
4	2405	A	C	C	C	C	G	G	T
5	2406	G	C	C	C	C	C	C	C
6	2407	A	C	C	C	C	G	G	T
7	2408	G	C	C	C	C	C	C	C
8	2410	G	C	C	C	C	G	G	T
9	2411	G	C	C	C	C	C	C	C
10	2412	A	C	C	C	C	C	C	C
11	2413	G	C	C	C	C	G	G	T
12	2414	A	C	C	T	C	G	G	T
13	2415	G	C	C	C	C	G	G	T
14	2416	A	C	C	T	C	G	G	T
15	2417	G	C	C	C	C	G	G	T
16	2418	G	C	C	C	C	C	C	C
17	2420	A	C	C	T	C	C	C	C
18	2421	G	C	C	C	C	G	G	T
19	2422	G	C	C	C	C	G	G	T
20	2423	A	C	C	T	C	G	G	T
21	2424	G	C	C	C	C	G	G	T
22	2425	G	C	C	C	C	G	G	T
23	2426	A	C	C	T	C	G	G	T
24	2427	G	A	T	C	T	C	C	C
25	2428	G	C	C	C	C	C	C	C
26	2429	A	C	C	T	C	G	G	T
27	2430	G	C	C	C	C	C	C	C
28	2431	G	C	C	C	C	C	C	C
29	2432	A	C	C	T	C	C	C	C
30	2433	A	C	C	C	C	C	C	C
31	2434	G	C	C	C	C	C	C	C
32	2435	G	C	C	T	C	C	C	C
33	2436	G	C	C	C	C	C	C	C
34	2437	G	C	C	C	C	G	G	T
35	2438	G	C	C	C	C	C	C	C
36	2439	G	C	C	C	C	G	G	T
37	2440	G	C	C	C	C	G	G	T
38	2441	G	C	C	C	C	G	G	T
39	2442	A	C	C	T	C	C	C	C
40	2444	G	C	C	C	C	C	C	C
41	2445	G	C	C	C	C	C	C	C
42	2446	G	C	C	C	C	C	C	C
43	2447	G	A	T	C	T	C	C	C
44	2448	G	C	C	C	C	G	G	T
45	2449	G	C	C	C	C	G	G	T
46	2450	G	C	C	C	C	C	C	C

^B^ The *ITS*, *psbA*, and *matK* SNP sites in the table correspond to the sites in the known sequences of KP711096.1, HQ600076.1, and AF164422.1 in Genbank, respectively.

## Appendix F

**Table A6:** Information on seed collection for the sample populations.

ID	Collection Time	City	County/District	Longitudes	Latitudes	Cultivation Mode
2401	16 September 2021	Yinchuan	Helan	106°30′35″ E	38°40′1″ N	one-crop system
2403	15 September 2021	Zhongwei	Zhongning	105°35′5″ E	37°28′11″ N	one-crop system
2404	16 September 2021	Yinchuan	Pingluo	106°19′17″ E	38°48′47″ N	one-crop system
2405	17 September 2021	Yinchuan	Helan	106°16′47″ E	38°43′53″ N	one-crop system
2406	16 September 2021	Yinchuan	Wuzhong	106°16′58″ E	38°12′27″ N	one-crop system
2407	17 September 2021	Shizuishan	Pingluo	106°19′17″ E	38°52′58″ N	one-crop system
2408	16 September 2021	Lingwu	Xindu Line	106°26′83″ E	38°08′05″ N	one-crop system
2410	16 September 2021	Yinchuan	Yimeng	106°39′59″ E	38°44′34″ N	one-crop system
2411	15 September 2021	Zhongwei	Zhongwei	105°42′6″ E	37°33′42″ N	one-crop system
2412	17 September 2021	Yinchuan	Helan	106°18′20″ E	38°45′40″ N	one-crop system
2413	15 September 2021	Zhongwei	Zhongwei	105°42′6″ E	37°33′42″ N	one-crop system
2414	16 September 2021	Yinchuan	Pingluo	106°44′0″ E	38°59′42″ N	one-crop system
2415	16 September 2021	Yinchuan	Helan	106°28′57″ E	38°35′40″ N	one-crop system
2416	17 September 2021	Yinchuan	Helan	106°11′53″ E	38°36′16″ N	one-crop system
2417	17 September 2021	Yinchuan	Helan	106°16′7″ E	38°40′50″ N	one-crop system
2418	16 September 2021	Yinchuan	Nandaigou	106°47′74″ E	38°46′34″ N	one-crop system
2420	16 September 2021	Shizuishan	Pingluo	106°35′42″ E	38°47′46″ N	one-crop system
2421	15 September 2021	Zhongwei	Shapotou	105°26′43″ E	37°28′4″ N	one-crop system
2422	15 September 2021	Zhongwei	Shapotou	105°26′43″ E	37°28′4″ N	one-crop system
2423	17 September 2021	Yinchuan	Helan	106°16′47″ E	38°43′53″ N	one-crop system
2424	15 September 2021	Zhongwei	Zhongwei	105°42′6″ E	37°33′42″ N	one-crop system
2425	14 September 2021	Yinchuan	Helan	106°16′47″ E	38°43′53″ N	one-crop system
2426	16 September 2021	Wuzhong	Mengjiazhuang	106°16′58″ E	38°12′27″ N	one-crop system
2427	16 September 2021	Yinchuan	Yimeng	106°39′59″ E	38°44′34″ N	one-crop system
2428	16 September 2021	Yinchuan	Helan	106°28′57″ E	38°35′40″ N	one-crop system
2429	17 September 2021	Yinchuan	Helan	106°11′53″ E	38°36′16″ N	one-crop system
2430	15 September 2021	Wuzhong	Litong	106°8′23″ E	37°52′24″ N	one-crop system
2431	16 September 2021	Shizuishan	Pingluo	106°35′42″ E	38°47′46″ N	one-crop system
2432	16 September 2021	Shizuishan	Pingluo	106°35′42″ E	38°47′46″ N	one-crop system
2433	16 September 2021	Yinchuan	Lingwu	106°26′83″ E	38°08′05″ N	one-crop system
2434	16 September 2021	Yinchuan	Dawukou	106°21′38″ E	38°52′44″ N	one-crop system
2435	16 September 2021	Shizuishan	Pingluo	106°31′36″ E	38°44′3″ N	one-crop system
2436	16 September 2021	Lingwu	Chongxing	106°17′19″ E	38°2′17″ N	one-crop system
2437	15 September 2021	Zhongwei	Zhongning	105°49′37″ E	37°31′43″ N	one-crop system
2438	16 September 2021	Shizuishan	Pingluo	106°31′36″ E	38°44′3″ N	one-crop system
2439	15 September 2021	Zhongwei	Shapotou	105°15′36″ E	37°28′26″ N	one-crop system
2440	15 September 2021	Zhongwei	Shapotou	105°15′36″ E	37°28′26″ N	one-crop system
2441	17 September 2021	Yinchuan	Helan	106°16′47″ E	38°43′53″ N	one-crop system
2442	16 September 2021	Yinchuan	Nandaigou	106°47′74″ E	38°46′34″ N	one-crop system
2444	15 September 2021	Zhongwei	Zhongning	105°35′5″ E	37°28′11″ N	one-crop system
2445	15 September 2021	Zhongwei	Zhongning	105°35′5″ E	37°28′11″ N	one-crop system
2446	16 September 2021	Shizuishan	Pingluo	106°44′0″ E	38°59′42″ N	one-crop system
2447	16 September 2021	Lingwu	Chongxing	106°17′19″ E	38°2′17″ N	one-crop system
2448	15 September 2021	Wuzhong	Litong	106°8′23″ E	37°52′24″ N	one-crop system
2449	15 September 2021	Zhongwei	Shapotou	105°15′36″ E	37°28′26″ N	one-crop system
2450	15 September 2021	Zhongwei	Shapotou	105°26′43″ E	37°28′4″ N	one-crop system

The populations collected in this study were all planted with single rice varieties in the same agricultural management measures and herbicide control programs.
